# Assessment and Treatment of Pediatric Sleep Problems: Knowledge, Skills, Attitudes and Practices in a Group of Community Child Psychiatrists

**DOI:** 10.3390/medsci6010018

**Published:** 2018-02-23

**Authors:** Ali Anwar, Michael D. Yingling, Alicia Zhang, Ujjwal Ramtekkar, Ginger E. Nicol

**Affiliations:** 1 Department of Psychiatry, Washington University School of Medicine, 660 S. Euclid Ave., Campus Box 8134, St. Louis, MO 63110, USA; s.anwar@wustl.edu (A.A.); yinglinm@wustl.edu (M.D.Y.); aliciazhang@wustl.edu (A.Z.); 2 Compass Health Network, University of Missouri Columbia School of Medicine, 3 Hospital Plaza Dr., Columbia, MO 65212, USA; uramtekkar@compasshn.org

**Keywords:** child psychiatry, sleep problems, medical education

## Abstract

As part of a university-based quality improvement project, we aimed to evaluate child psychiatrists’ knowledge, skills, attitudes, and practices regarding assessment and treatment of pediatric sleep problems. We developed a nine-question survey of knowledge, skills, attitudes, and practices regarding assessing for and treating sleep complaints in pediatric patients, and administered this survey to child psychiatrists in training and in practice in the state of Missouri. Respondents reported sleep hygiene as the first-line treatment strategy, followed by the use of supplements or over-the-counter remedies. The most common barriers to evidence-based assessment and treatment of sleep problems were the lack of ability to obtain reliable history, and parental preference for medications over behavioral approaches for sleep concerns. These results suggest potential opportunities for enhancing knowledge regarding validated assessment tools and non-pharmacological treatment options for sleep problems. Additional research is needed to further assess the quality and type of sleep education provided in child psychiatry training programs.

## 1. Background

Sleep problems are a common complaint amongst individuals suffering from psychiatric illness [1,2,3]. There has been emerging evidence in recent years about the complex relationship between sleep and psychiatric disorders that has suggested the existence of a bidirectional relationship [4]. Sleep is part of the symptom criteria in the Diagnostic and Statistical Manual of Mental Disorders, Fifth Edition (DSM-V) for numerous major psychiatric conditions, including those first observed in childhood. Sleep disorders are more common in youth who may attempt suicide or engage in high-risk self-harming behavior [5,6]. Even in psychiatric disorders where sleep disruption is not a major symptom marker, sleep is still thought to play a role in the development and maintenance of dysfunctional symptoms. For example, attention deficit hyperactivity disorder ADHD symptoms are commonly presented complaints in outpatient child psychiatric practices. Children with ADHD can have more bedtime resistance, more issues with initiation of sleep, more nighttime awakenings, difficulties with morning awakenings, sleep-disordered breathing, and daytime sleepiness [7]. Sleep apnea itself is associated with symptoms of hyperactivity, impulsivity, inattention, and poor academic performance [8,9]. Studies have suggested that there may be a higher prevalence of restless legs syndrome and periodic limb movement syndrome in children with ADHD [10]. Autism spectrum disorder is another condition where sleep is not part of the symptom criteria, but sleep problems are common. An estimated 40–80% of children with autism spectrum disorder have sleep problems [11]. Sleep disturbances are common in major mood disorder diagnoses, from decreased need for sleep in bipolar mood disorders to hypersomnia or varying degrees of insomnia in major depression. Finally, insomnia is a frequent complaint in schizophrenia, and can have negative effects on quality of life and cognition. It has even been suggested that sleep deficits may be a precursor of psychosis [12]. Many psychiatric medications that are often prescribed can have an impact on sleep as well. Selective serotonin reuptake inhibitors (SSRIs) are among the most commonly used medications to treat depression and anxiety, and have been known to cause restless legs syndrome or periodic limb movements [13].

Despite the importance of sleep in day-to-day functioning and development, and the role that sleep plays in the development and maintenance of psychiatric conditions, many physicians across various specialties have limited education on adequately screening, diagnosing, and treating sleep problems. In 2001, Owens et al. surveyed 828 physicians on their knowledge about sleep disorders in the pediatric population. Most of the surveyed physicians were primary care physicians, about 75% of them being pediatricians. Less than half of the participants responded that they felt confident in screening for sleep problems in children. Only 25% of participants felt confident in treating sleep in children. The authors of this study felt that this may be due to gaps in basic knowledge about pediatric sleep disorders. They referenced prior studies that documented the limited amount of didactics and other teaching on pediatric sleep disorders in medical school and in residency. The lack of emphasis on sleep issues in pediatric textbooks was also mentioned as a contributor to the gaps in knowledge [14].

Adding to challenges in treating pediatric sleep complaints is the limited data supporting the use of prescription medications for this indication in children. In 2010, Stojanovski et al. looked at trends in medications prescribed for sleep problems in US pediatric outpatients. The survey included 35% pediatricians, 24% psychiatrists, 13% general/family practice physicians, 4% neurologists, and 23% other specialists. They found that more than 80% of visits where sleep problems were reported led to the use of a prescription medication. Furthermore, medications that physicians used to treat sleep problems were those primarily used in psychiatric conditions and had limited evidence for safety and efficacy in children [15]. Despite the prevalence of sleep complaints in children with psychiatric disorders, and the common use of psychotropic medications to treat pediatric sleep problems, there remains a paucity of research assessing the practices and preferences of pediatric psychiatrists.

Given the prominence of sleep disturbances in children who come to clinical attention for psychiatric complaints, we aimed to assess clinician practices in this population in our region. We aimed to evaluate current knowledge, skills, attitudes, and practices across a range of clinician experience related to assessment and treatment of sleep problems in the pediatric psychiatric population. Our goal was to identify potential areas for intervention and/or further education of physicians to improve current practice.

## 2. Materials and Methods

### 2.1. Knowledge, Skills, Attitudes and Practices Survey

We developed a simple nine-question survey (see Supplementary materials) as part of a quality improvement project conducted at Washington University School of Medicine between January and June 2017, to evaluate psychiatric clinician knowledge, skills, attitudes, and practices regarding assessing for and treating sleep complaints in pediatric patients. Questions were generated based on a review of existing literature regarding current guidelines [16,17], community provider knowledge and practices, and the research team’s previous experience working with pediatric sleep disorders [18]. All survey responses were anonymous.

### 2.2. Participants

The questionnaire was distributed in person at a regional child psychiatry organization meeting (Greater St. Louis Area Regional Organization for Child & Adolescent Psychiatry, GSL ROCAP), as well as to general psychiatry and child psychiatry trainees at Washington University School of Medicine. GSL ROCAP members unable to attend meetings in person were emailed the survey.

### 2.3. Statistical Analysis

Data were analyzed using SPSS software (v.22; IBM Corp., Armonk, NY, USA). All available data from all participants were used. Descriptive statistics (mean, frequencies, and proportions) were generated for survey responses. Likert scale items were converted from text value to numerical rating (e.g., 1 = not at all; 3 = very much). Responses to each survey question are reported as number (*n*) and percentage of clinicians with a response in each category for a given question.

## 3. Results

A total of 55 clinicians (31.6%) out of 174 contacted responded to the survey. Respondents were 1–5 years in practice (*n* = 4, 7.3%), 6–10 years in practice (*n* = 7, 12.7%), and 10–15 years in practice (*n* = 11, 20.0%). Most participants practiced in an outpatient setting: 61.8% (*n* = 34) reported working in an academic outpatient clinic; 49.1% (*n* = 27) reported working in a community mental health clinic; and 23.6% (*n* = 13) reported working in a private outpatient clinic. Note that participants were able to select multiple clinical settings if they worked in more than one.

**Figure 1:**
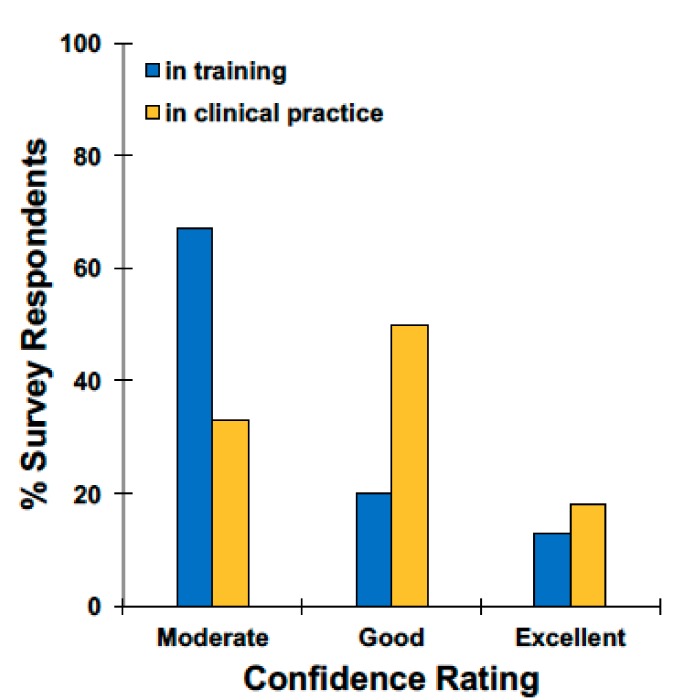
Respondent self-report of confidence in assessing pediatric sleep concerns by level of clinical experience.

The majority of participants (*n* = 50, 90.9%) reported that they assess for sleep problems in all of their patients, with 78.6% (*n* = 44) reporting that they assess for sleep problems at every clinical visit. When participants were asked to rate their confidence in their ability to assess sleep problems on a scale of 1–5, with 1 being poor confidence and 5 being excellent confidence, 41.8% (*n* = 23) chose 3 (moderate confidence), 41.8% (*n* = 23) chose 4 (good confidence), and 16.4% (*n* = 9) chose 5 (excellent confidence). In total, 72.2% (*n* = 13) of participants who have been in practice for at least 15 years said they had good or excellent confidence (4 or 5) in their ability to assess sleep problems. Only 33.3% (*n* = 5) of participants still in training said they had good or excellent confidence (4 or 5) in their assessment of sleep problems (Figure 1).

One survey participant reported using a specific sleep questionnaire, while the overwhelming majority (*n* = 54, 98.2%) reported assessing for sleep problems as part of their clinical interview. Difficulty in obtaining accurate information from the patient or adult caregiver was reported as a barrier to assessing sleep problems by 45.5% (*n* = 25) of respondents; 41.8% (*n* = 23) reported limited time during the clinical visit as a major barrier to obtaining accurate information regarding sleep problems.

**Figure 2:**
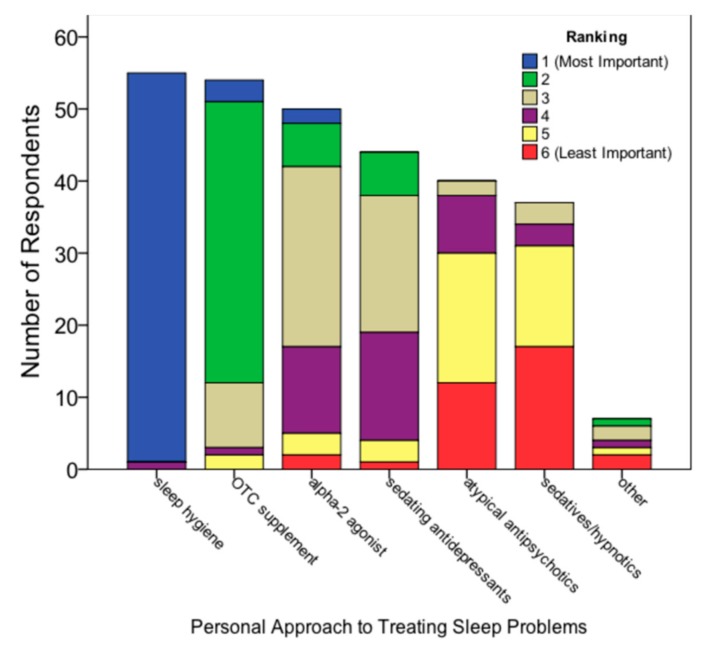
Ranked importance of clinical approaches to sleep problems.

When asked which treatment strategy (sleep hygiene, alpha-2 agonists, over-the-counter supplements, atypical antipsychotics, sedatives/hypnotics, sedating antidepressants, or other) was viewed as most important, almost all participants (*n* = 54, 98.2%) ranked sleep hygiene as most important. Participants still in training (*n* = 15) all ranked sleep hygiene as most important. Over-the-counter supplements (e.g., melatonin, valerian) were most-ranked second (*n* = 39, 70.9%) and alpha-2 agonists (clonidine and guanfacine) were most-ranked third (*n* = 25, 45.5%). Participants still in training ranked over-the-counter supplements as second most important (*n* = 12, 80%) compared with 67.5% (*n* = 27) of participants who had completed training (Figure 2).

Anticipated non-compliance with recommendations (52.7%, *n* = 29) and patient/guardian preference for medications (32.7%, *n* = 18) were the most selected reasons for barriers to using sleep hygiene as a first-line treatment for sleep problems. 

## 4. Discussion

To our knowledge, this is the first study to report on the skills, attitudes, and practices of child psychiatric providers regarding pediatric sleep concerns. A better understanding of clinician perspectives on this matter is important in identifying knowledge gaps and developing educational strategies for improving adherence to best practices. While the majority of our respondents reported routinely assessing for sleep concerns in their patients, they also noted that major barriers to assessment were limited time and difficulty in obtaining accurate information. Despite this rather universal report of challenges in assessment, only one respondent reported routinely using a validated sleep assessment tool to screen for sleep problems. The majority of our respondents also reported first-line use of low-risk treatment approaches with modest evidence, including education on sleep hygiene and recommending use of over-the-counter supplements such as melatonin. However, the importance of other treatment approaches was variably ranked, with no respondents reporting use of non-pharmacological strategies other than sleep hygiene. In addition, while nearly all respondents ranked sleep hygiene as being “very important” in their treatment approach, patient/guardian preference for medications and anticipated non-compliance were reported as significant barriers to using it as a first-line intervention. Finally, we observed a difference in confidence and knowledge based on number of years in practice, with more seasoned clinicians having greater confidence in assessment abilities. These results suggest potential opportunities for educating psychiatric providers on assessment tools and behavioral approaches for sleep problems in children.

Limited time for assessment and difficulty obtaining accurate information were identified as the biggest barriers by those surveyed in this study. Although clinical screening for sleep difficulties cannot take the place of gold standard sleep assessments, particularly for the evaluation of disorders such as obstructive sleep apnea, validated sleep questionnaires completed by the patient/guardian in the waiting room prior to the visit may address barriers to screening reported by our respondents. Validated self-report questionnaires commonly used in the pediatric sleep assessment include the Pediatric Sleep Questionnaire (PSQ), the Pediatric Daytime Sleepiness Scale (PDSS), the BEARS (B = Bedtime Issues, E = Excessive Daytime Sleepiness, A = Night Awakenings, R = Regularity and Duration of Sleep, S = Snoring), and the Ten Item Sleep Screener. The PSQ is a 49-item questionnaire for parents that is divided into behavioral, sleepiness, and snoring domains. This can be used for patients aged 2–18 years. It has sensitivity and specificity of 0.81 and 0.87, respectively [19]. The PDSS is an eight-item questionnaire where items are scored on a Likert scale rating system. It is used to measure excessive sleepiness in children and has been shown to have good internal consistency [20]. The BEARS pediatric sleep screening tool assesses sleep disorders in children aged 2–18 years. It has parent-directed and child-directed questions that evaluate five domains including bedtime problems, excessive daytime, awakenings during the night, regularity and duration of sleep, and snoring. Questions are distinct for toddlers/preschoolers (2–5 years), school-aged children (6–12 years), and adolescents (13–18 years) [21]. Another pediatric sleep screening tool is the Ten Item Sleep Screener which asks such questions as “Does the child snore lightly or loudly at night?”; “Does the child wake up frequently in the night?”; “Does the child have a difficult temperament (irritable or easily frustrated)?” [22]. Additional studies evaluating the validity of these screening tools compared with gold standard assessments would be helpful in this population.

The fact that respondents ranked pharmacological interventions as having limited and varying importance in their clinical practices is likely a reflection of the lack of evidence supporting the use of medications for the treatment of insomnia and other sleep-related problems in children [1]. Participants in our survey overwhelmingly ranked sleep hygiene as the most important intervention in addressing sleep problems, but noted important barriers to using it consistently as a first-line treatment approach. Cognitive behavioral therapy for insomnia (CBT-I) is a structured behavioral intervention that has been shown to be effective and have fewer risks than medication [23], but was not listed by any respondents as a primary treatment strategy. This suggests a limited knowledge of or ability to provide evidence-based behavioral treatments such as CBT-I, which could be usefully included in psychotherapy education during training.

Although not directly addressed by our survey, others have posited that low confidence in and knowledge of treatment for pediatric sleep problems—particularly in child psychiatry trainees—is related to the lack of a universally-adapted pediatric sleep curriculum in residency training [18]. In 2002, Krahn et al. surveyed 98 program directors of general psychiatry residencies in the USA, and observed a lack of standardized approaches to sleep education in training programs. They reported that 82% of participating programs had sleep lectures as part of didactics. However, only 44% offered a sleep medicine elective, and the majority did not have a faculty member that was a sleep medicine specialist [24]. Similarly, Khawaja et al. surveyed 39 chief psychiatry residents, and found that about 90% reported that their training programs offered didactic sleep education. However, only 38% of programs had faculty that were trained in sleep medicine, and only 34% offered a sleep medicine elective [25]. No programs reported a structured curriculum devoted specifically to pediatric sleep concerns. These findings highlight the need to further study the effectiveness of current sleep education approaches for psychiatric trainees, with a focus on those going into pediatric psychiatry.

This study was subject to important limitations. In particular, this was a small quality improvement project aiming to assess clinical practices within the Eastern Missouri region, and may not be generalizable to practitioners in other regions. Most of the participants were either in training or had been in practice for 15 years or more, with low representation of early career psychiatrists (1–5 years in practice). Additionally, we did not assess current training practices nor query respondents on their educational experiences during training with respect to assessment and treatment of pediatric sleep concerns. Finally, we did not specifically assess for knowledge of psychotherapeutic approaches to pediatric sleep problems. A larger sample size with a better representation of clinicians at different stages of their careers, assessing for training experiences, and knowledge of behavioral approaches other than sleep hygiene is necessary to further assess for knowledge gaps and develop targeted educational approaches for pediatric sleep training in child psychiatry training. However, our study did provide some insight into potential areas for quality improvement in assessment of sleep problems and future directions for research and clinician education. 

## Supplementary Materials

The following are available online at http://www.mdpi.com/2076-3271/6/1/18/s1, Sleep Problems Survey.
