# Supplementary material for: Assessment and Treatment of Pediatric Sleep Problems: Knowledge, Skills, Attitudes and Practices in a Group of Community Child Psychiatrists
Source: Med Sci (Basel). 2018 Feb 23;6(1):18. doi: 10.3390/medsci6010018 (PMC5872175; doi:10.3390/medsci6010018)
Supplement: Supplementary file 1 [file medsci-06-00018-s001.pdf]

Sleep Problems Survey

The purpose of this survey is to evaluate current community practices related to clinical assessment and treatment of sleep problems.

Firstly, we would like some information about you and where you practice.

1) How many years have you been in practice?
□ still in training
□ 1–5
□ 6–10
□ 10–15
□ 15+

2) Practice location (select all that apply)
□ academic outpatient clinic
□ academic hospital inpatient
□ private hospital inpatient
□ private outpatient clinic
□ community mental health clinic
□ residential facility
□ PHP/IOP
□ other:_____________

Now, we would like to know about your practice with regard to assessment of sleep problems.

3) Do you assess for sleep problems in all your patients?
□ Yes
□ No

4) How often do you assess for sleep problems in your patients?
□ every appointment
□ every 6 months
□ every year
□ never
□ other: ____________

5) How confident are you in your abilities to assess for sleep problems in your patients? (Rate on a scale of 1-5.)
□ 1 (Poor)
□ 2
□ 3

□ 4
□ 5 (Excellent)

6) How do you assess for sleep problems in your patients?
   □ as part of clinical interview
   □ review of systems form
   □ specific sleep questionnaire
   □ only if patient reports sleep problems
   □ other: ________________________

7) What do you think is the biggest barrier to routinely assessing for sleep problems in your patients?
   □ limited time
   □ difficulty obtaining accurate information
   □ lack of effective treatments
   □ lack of knowledge about recommended treatments
   □ other: _________________________________

Finally, we would like to know about your practice with regard to treatment of sleep problems.

8) Please rank the items below in descending order of importance (1 is very important, 6 is the least important) to your personal approach to treating sleep problems.
   *if more than one approach at the same time, please rank each choice with the same number
   *if a choice is not part of your approach, please leave blank

   __ sleep hygiene
   __ alpha-2 agonists (e.g., clonidine)
   __ OTC supplement (e.g., melatonin or valerian)
   __ atypical antipsychotics (e.g., quetiapine)
   __ sedatives/hypnotics (e.g., clonazepam or zolpidem)
   __ sedating antidepressants (e.g., trazodone or mirtazapine)
   __ other (please specify)_________________________

9) What do you feel is the biggest barrier to using sleep hygiene as a first-line treatment for sleep problems?
   □ takes too much time to discuss
   □ lack of knowledge about sleep hygiene
   □ anticipated patient/guardian compliance with recommendations
   □ patient/guardian preference for medications
   □ Other: ____

Thank you for participating in this survey. If you have any questions, comments, or suggestions please contact Ali Anwar at ***s.anwar@wustl.edu****.*
